# Supplementary material for: Causal interplay between lactose intolerance and gut microbiota: a combined bidirectional Mendelian randomization and in vivo validation study
Source: Front Nutr. 2026 Jun 1;13:1803337. doi: 10.3389/fnut.2026.1803337 (PMC13265576; doi:10.3389/fnut.2026.1803337)
Supplement: Supplementary file 1 [file Data_Sheet_1.zip › supplementary materials/Forward/leave one out cross validation/ebi-a-GCST90027542.finngen_R12_E4_LACTONAS.pdf]

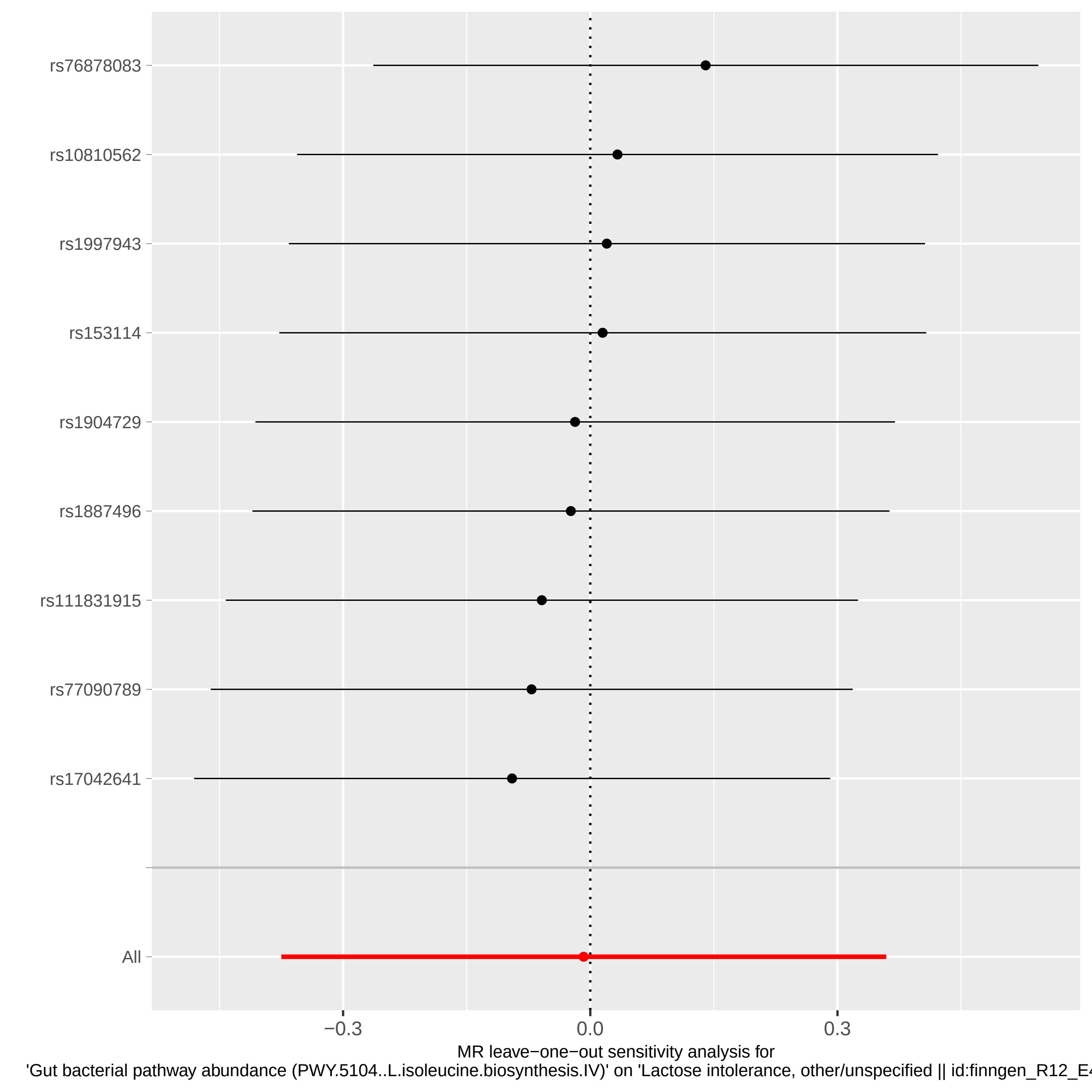

rs76878083
rs10810562
rs1997943
rs153114
rs1904729
rs1887496
rs111831915
rs77090789
rs17042641
All
−0.3
0.0
0.3
MR leave−one−out sensitivity analysis for
'Gut bacterial pathway abundance (PWY.5104..L.isoleucine.biosynthesis.IV)' on 'Lactose intolerance, other/unspecified || id:finngen_R12_E4
